# Supplementary material for: Dynalign II: common secondary structure prediction for RNA homologs with domain insertions
Source: Nucleic Acids Res. 2014 Nov 21;42(22):13939–48. doi: 10.1093/nar/gku1172 (PMC4267632; doi:10.1093/nar/gku1172)
Supplement: SUPPLEMENTARY DATA [file supp_gku1172_nar-02021-z-2014-File012.zip › manual/javadoc/RNAstructure_java_drawing/source/runners/RunnerTemplate.html]

RunnerTemplate


JavaScript is disabled on your browser.


- Overview
- Package
- Class
- Tree
- Index
- Help

- Prev Class
- Next Class

- Frames
- No Frames

- All Classes

- Summary:
- Nested |
- Field |
- Constr |
- Method

- Detail:
- Field |
- Constr |
- Method


RNAstructure\_java\_drawing.source.runners

## Class RunnerTemplate

- java.lang.Object
- - RNAstructure\_java\_drawing.source.runners.RunnerTemplate

- Direct Known Subclasses:
  :   PlotRunner, StructureRunner

  ---

    

  ```
  public abstract class RunnerTemplate
  extends Object
  ```

  A class that templates the way in which the RNAstructure Java drawing
  framework is run independently.

- - ### Constructor Summary

    Constructors

    Constructor and Description

    `RunnerTemplate(String libraryName)`

    Constructor.
  - ### Method Summary

    Methods

    | Modifier and Type | Method and Description |
    | --- | --- |
    | `void` | `addClosingListener(JDialog dialog)` Add a listener to the dialog spawned by this template that exits the application when the dialog is closed. |
    | `protected abstract String` | `selectFile()` Select an image file. |

    - ### Methods inherited from class java.lang.Object

      `clone, equals, finalize, getClass, hashCode, notify, notifyAll, toString, wait, wait, wait`

- - ### Constructor Detail


    - #### RunnerTemplate

      ```
      public RunnerTemplate(String libraryName)
      ```

      Constructor.

      Parameters:
      :   `libraryName` - The name of the native library connected to this
          template.
  - ### Method Detail


    - #### addClosingListener

      ```
      public void addClosingListener(JDialog dialog)
      ```

      Add a listener to the dialog spawned by this template that exits the
      application when the dialog is closed.

      Parameters:
      :   `dialog` - The dialog to close.


    - #### selectFile

      ```
      protected abstract String selectFile()
      ```

      Select an image file.

      Returns:
      :   The image file.


- Overview
- Package
- Class
- Tree
- Index
- Help

- Prev Class
- Next Class

- Frames
- No Frames

- All Classes

- Summary:
- Nested |
- Field |
- Constr |
- Method

- Detail:
- Field |
- Constr |
- Method
